# Metabolic Phenotyping Identifies Responders to Sustainable Healthy Diets: Analysis from the MyPlanetDiet Randomized Controlled Trial

**DOI:** 10.1016/j.cdnut.2026.109510

**Published:** 2026-08-10

**Authors:** Katie P Davies, Eileen R Gibney, Ursula M Leonard, Leona Lindberg, Jayne V Woodside, Mairead E Kiely, Anne P Nugent, Elena Arranz, Marie C Conway, Sinead N McCarthy, Aifric M O’Sullivan

**Affiliations:** 1 Institute of Food and Health, School of Agriculture and Food Science, University College Dublin, Dublin, Ireland; 2 Cork Centre for Vitamin D and Nutrition Research, School of Food and Nutritional Sciences, University College Cork, Cork, Ireland; 3 Centre for Public Health, School of Medicine, Dentistry and Biomedical Sciences, Queen's University Belfast, Belfast, Northern Ireland, United Kingdom; 4 Institute for Global Food Security, School of Biological Sciences, Queen’s University Belfast, Belfast, United Kingdom; 5 Instituto de Investigación en Ciencias de la Alimentación, CIAL (CSIC-UAM, CEI UAM+CSIC), Universidad Autónoma de Madrid, Madrid, Spain; 6 School of Biological, Health and Sports Sciences, Technological University Dublin, Dublin, Ireland; 7 Department of Agrifood Business and Spatial Analysis, Teagasc Food Research Centre, Dublin, Ireland

**Keywords:** interindividual variation, metabolic health, metabolic phenotype, metabolomics, sustainable healthy diets

## Abstract

**Background:**

Understanding interindividual variation is critical for developing strategies to improve health. Metabolic phenotypes impact how individuals respond to dietary change. Although literature has examined responses to dietary interventions between metabolic phenotypes, none have yet considered sustainable healthy diets.

**Objectives:**

Exploratory, hypothesis-generating, post hoc secondary analysis from the MyPlanetDiet randomized controlled trial is used to examine subgroup response to sustainable healthy diets. The aim is to identify metabolic phenotype subgroups (MPs) within the MyPlanetDiet sample population and examine if MPs responded differently to dietary change.

**Methods:**

The MyPlanetDiet study was a 12-wk randomized controlled trial testing the impacts of more sustainable healthy diet advice relative to advice from food-based dietary guidelines. Dietary assessments, fasting serum samples, and anthropometry were collected at baseline and endpoint. *k*-means cluster analysis of baseline serum metabolic health markers identified MPs. Metabolomic profiles, dietary intakes, and changes in metabolic health over time were compared between MPs using partial least squares discriminant analysis and analysis of covariance.

**Results:**

Three distinct MPs were identified with significantly different metabolomic profiles despite comparable baseline dietary intakes. Metabolic phenotype 1 (MP1) was categorized as the most metabolically unhealthy, metabolic phenotype 2 (MP2) was mostly female with the lowest anthropometry and high total cholesterol, and metabolic phenotype 3 (MP3) was the youngest with the healthiest blood lipid concentrations. There were significant time × group interactions for total cholesterol (*P* = 0.001), high-density lipoprotein cholesterol (*P* < 0.001), triglycerides (*P =* 0.002), and C-reactive protein (*P =* 0.043) between MPs. Significant decreases in total cholesterol within MP1 (5.92 ± 0.88 mmol/L to 5.74 ± 0.83 mmol/L, *P =* 0.006) and MP2 (5.81 ± 0.86 mmol/L to 5.64 ± 0.94 mmol/L, *P* = 0.018) were observed, but no significant difference was observed in MP3 (*P* = 0.146). Increased intakes of fruit and vegetables and whole grains were associated with decreased total cholesterol in MP1 and MP2 (all *P <* 0.05).

**Conclusions:**

Metabolic phenotyping shows interindividual variation in metabolic response to dietary change. Metabolic phenotyping could be a valuable tool for informing personalized nutrition strategies to improve diet and metabolic health.

This trial was registered at clinicaltrials.gov as NCT05253547.

## Introduction

Higher diet quality is associated with reduced risk of chronic disease and mortality [1]. However, increasing diet quality in randomized controlled trials (RCTs) leads to mixed impacts on health markers, such as blood lipids, anthropometry, or blood pressure [[2], [3], [4]]. Interindividual variation and metabolic response to interventions are underpinned by an individual’s metabolic phenotype. Metabolic phenotypes are shaped by genetics, environment, gut microbiome, age, sex, diet, and body composition [5,6]. For example, Kirwan et al. [7] analyzed baseline traits linked to cholesterol response in the Food4Me RCT, identifying factors such as age, glucose concentrations, and fatty acid concentrations as predictors of cholesterol response. These traits help define metabolic phenotypes that can predict future intervention responses. Grouping individuals by metabolic phenotype can therefore be an exploratory tool for identifying responders and nonresponders in dietary interventions. As a hypothesis-generating analysis, this work has the potential to identify novel targets for more personalized dietary interventions that may improve health outcomes by accounting for individual variation in response to dietary change.

Metabolic phenotype–based personalized nutrition feedback systems represent a promising strategy for improving health through dietary change. Personalized nutrition provides individualized dietary recommendations based on personal characteristics to optimize health outcomes [2]. Evidence supports personalized nutrition as a useful and scalable tool to support dietary change. Studies have investigated multiple levels of personalization, ranging from conventional dietary advice to recommendations informed by individual phenotypic and biological characteristics, to evaluate their effectiveness [2,4,8]. As an example, researchers have built dietary feedback tailored to support the health of a particular metabolic phenotype subgroup (MP), such as offering cholesterol-lowering dietary advice to those with elevated concentrations of total cholesterol (total-C) [9]. For example, O’Donovan et al. [9] grouped individuals into MPs based on their baseline total-C, HDL cholesterol, triglycerides (TAGs), and glucose and developed dietary feedback tailored to each MP. O’Donovan et al. [9,10] showed strong agreement between MP-derived dietary advice, advice from a dietitian, or the standardized personalized advice from the Food4Me project. A similar MP-based feedback structure was later used in a dietary intervention, which led to improved metabolic health and diet quality [8,11]. Although such evidence is promising for the future of improving health, future personalized nutrition strategies must work in conjunction with wider nutrition-related goals to support and promote all dimensions of health. When looking at the current state of nutrition science, it is inevitable that future nutrition-related strategies will be influenced by the need to reduce the environmental impact of our food systems [12]. Mounting environmental pressure and instability increase risk of nutrition-related poor health and underscore the urgent need to prioritize the transition to sustainable healthy diets [12,13]. Therefore, it will be important to examine whether sustainable healthy diets will impact metabolic health, and furthermore, if such recommendations could or should be tailored to specific subgroups. To our knowledge, there have been no published interventions using a metabolic phenotype–based approach to support the transition to a more sustainable healthy diet. In one of the few published RCTs testing personalized sustainable healthy dietary advice, we showed that MyPlanetDiet participants in the intervention and control groups reduced mean diet-related greenhouse gas emissions (GHGE) and increased mean diet quality score during the study [14]. Therefore, we concluded that both personalized advice for sustainable healthy diets and personalized advice on Healthy Eating Guidelines led to more sustainable and healthy diets in our sample population. On completion of the MyPlanetDiet study, the findings present an opportunity to investigate whether metabolic phenotype–based personalization could or should be applied in future studies. Using data from MyPlanetDiet, we offer an exploratory, hypothesis-generating, proof-of-concept, post hoc secondary analysis to inform future personalized nutrition interventions for sustainable healthy diets. An essential first step is to determine whether individuals differ in their metabolic responses to sustainable healthy diets and to identify the key metabolic traits that may inform future MP-based personalized nutrition interventions. The aim of this secondary research is to identify MPs using the MyPlanetDiet cohort and examine whether MPs responded differently to dietary change.

## Methods

### MyPlanetDiet study design and participants

The detailed MyPlanetDiet protocol and study findings have been reported elsewhere [14,15]. An overview of the MyPlanetDiet methodology will be presented here with the description of the present analysis. The MyPlanetDiet RCT was a 12-wk single-blinded parallel trial testing the impact of following a sustainable healthy diet on diet-related GHGE. Individuals were randomly assigned to receive personalized advice based on a sustainable healthy diet (intervention) or based on Healthy Eating Guidelines (control). The study ran from March 2022 to May 2023 at three universities on the island of Ireland, at which all corresponding Research Ethics Committees provided ethical approval or exemption {University College Dublin [LS-21-51-Davies-O’Sullivan], University College Cork [ECM 4 (cc) 10/8/2021 and ECM 3 (f) 19/10/2021], and Queen’s University Belfast [MHLS_21_109]}. The study was registered at clinicaltrials.gov (NCT05253547). All study procedures were carried out according to a centralized protocol and were in line with the Declaration of Helsinki.

The study aimed to recruit 360 participants (120 per site) based on 20% difference in diet-related GHGE between control and intervention group diets at the end of the study (80% power, 5% significance). Healthy adults aged 18 to 64 y who were following moderate-to-high greenhouse gas–emitting diets (self-reported intake of beef or lamb ≥3 times per week) were eligible to take part in the study. Individuals who had medical conditions, medication use, or supplement use that interfered with study outcomes were excluded from taking part. Eligible participants signed informed consent and were randomly assigned using site-specific blocked randomization lists and stratified by age and sex. Participants were blinded to their study group.

### Dietary and lifestyle assessment

After randomization, participants completed dietary assessments and a health and lifestyle questionnaire using Foodbook24. Foodbook24 is an online 24-h recall tool that has been previously validated and described [16,17]. Dietary assessments included three 24-h recalls on nonconsecutive days and were collected at baseline (week 0) and endpoint (week 12). Dietary intake data derived from McCance and Widdowson’s Composition of Foods Integrated Dataset and the Irish Food Composition Database were used to calculate mean daily nutrient and food group intakes [16]. Individuals were provided with personalized advice based on their baseline dietary assessment data. More details on dietary intake assessments and the personalized feedback can be found in the study protocol [15]. Baseline dietary intakes and changes in food group intake over time were analyzed.

### Metabolic health data collection

The study included two onsite visits (baseline and endpoint) to a study center. At each study visit, fasting anthropometry, clinical measurements, and serum samples were collected in accordance with study standard operating procedures. Serum tubes were inverted five times after collection and left at room temperature for 30 min. Serum was then centrifuged for 15 min at 4°C and 1500 × *g* before being aliquoted and stored at −80°C until analysis.

## Clinical chemistry analysis

Serum samples were used to measure concentrations of total-C, LDL cholesterol, HDL cholesterol, TAGs, glucose, insulin, and C-reactive protein (CRP). Analysis was carried out in the Mater Misericordiae University Hospital (MMUH) (Dublin, Ireland) using Alinity c Clinical Chemistry Analyser (Abbott Laboratories) and the corresponding reagent kits. MMUH is an Irish National Accreditation Board laboratory that operates in compliance with International Standard ISO 15189 and the European Union Blood Directive 2002/98/EC. Internal quality controls aligned with manufacturer guidelines were used to monitor quality and accuracy. Per hospital protocol, LDL cholesterol was directly measured. If TAG concentration was <2.2 mmol/L, LDL cholesterol was calculated using Friedewald’s equation. The lowest limit of detection for CRP was 0.4 mmol/L. If CRP levels were below this threshold, the median between zero and the lowest level of detection was used instead.

### Metabolomic analysis

Serum samples were analyzed in the Conway Institute of Biomolecular and Biomedical Research in University College Dublin. Targeted metabolomic analysis was performed with Biocrates AbsoluteIDQ p180 kits (Biocrates). Samples were prepared per manufacturer guidelines and as previously described [18]. Briefly, a Sciex QTRAP 6500+ coupled to a Sciecx ExionLC series UHPLC capability was used for analysis. There were 131 metabolites identified and quantified, including 21 amino acids, 10 biogenic amines, 1 hexose, 7 acylcarnitines, 10 lysophosphatidylcholines, 14 sphingomyelins (SMs), and 68 phosphatidylcholines (PCs), of which 33 were diacyl (aa), and 35 were acyl-alkyl (ae). Amino acids and biogenic amines were measured using liquid chromatography–mass spectrometry. The remaining metabolites were analyzed using flow injection analysis tandem mass spectrometry. The quantification of metabolites was performed using internal standards. Data quality was evaluated by testing quality control samples. Only metabolites where >75% of samples were above the limit of detection were included in the data. Metabolite concentrations are reported in micromoles.

### Identifying metabolic phenotypes

*k*-means clustering was used to group individuals based on baseline concentrations of total-C, HDL cholesterol, TAGs, and glucose (standardized using *z*-scores), selected based on previous research [[8], [9], [10], [11],^19^]. *k*-means exclusively groups individuals with similar characteristics within a designated number of clusters. The first iteration of clustering assumed three clusters. One cluster (*n* = 2) was categorized as outliers due to high glucose concentrations (*z* = 10). A second iteration of *k*-means clustering was performed with four clusters. The same outlier cluster (*n* = 2) remained and was therefore excluded from the analysis. After removing the outlier cluster, the number of clusters was validated using the elbow method and gap statistic (Supplemental Figure 1). The three remaining metabolic phenotypes were used in the present analysis. The MPs were used to assess differences in markers of metabolic health, dietary intakes, and metabolomic profiles at baseline. Further analysis sought to determine whether changes in food group intakes over time impacted metabolic health markers between MPs differently. Changes in food group intake were analyzed using the difference from baseline to endpoint for each group. For each food group, tertiles were used to assess whether food group changes were associated with a change in biomarkers. Food groups such as fruit and vegetable intake, whole grain intake, and meat intake were separated into low, medium, and high change. The change in these food groups was almost all in one direction, and therefore the tertiles represent the magnitude of change. The directional change reflects sustainable healthy dietary advice; for example, with fruit and vegetables and whole grains, those with “high” change had the largest increases in their intakes during the study. For meat intake, a high change corresponds to the largest decrease in meat intake. When examining dairy, however, there was more variation with participants both increasing and decreasing dairy intake during the study. The tertiles of change for dairy are instead separated into increasers, maintainers, and decreasers. To examine how dietary change affected metabolic health biomarkers, total-C was selected because of its previous use as a marker of metabolic health [7] and because of the change over time presented later in this analysis.

### Statistical analysis

IBM SPSS Statistics (v29) for Macintosh (IBM Corp.) and R Studio (v4.5.1) (R Core Team) were used for statistical analysis. Data are presented as mean ± SD to 2 decimal places. Continuous variables were assessed for normality, and nonnormally distributed variables were transformed into normality using logarithmic or square root functions. For all parametric tests, Pearson’s correlation was used to determine appropriate covariates. Covariates, including sex, age, BMI, and energy intake, were assessed for all dependent variables reported. Significant covariates were retained in the final statistical models and included as adjustment variables to account for potential confounding effects on the associations of interest. Differences in intervention group distribution among MPs were assessed using chi-square tests. Baseline metabolic health data, metabolites, food group intakes, and nutrient intakes were compared between MPs using univariate general linear model analysis. Principal component analysis showed separation; therefore, partial least squares discriminant analysis (PLS-DA) was conducted to assess differences in metabolomic profiles between MPs. All metabolites were adjusted for age and sex before PLS-DA. Variable Importance in Projection (VIP) scores for each PLS-DA model were examined to explore the metabolites differentiating the phenotypes from one another. VIPs >1.2 were considered of interest. PLS-DA models were validated using 5-fold cross-validation, in which 80% of the data were used to predict the remaining 20% in five iterations. Models were assessed using *R*^2^, *Q*^2^, and permutation tests (*n* = 1000), where *R*^2^ represents the proportion of variance within the metabolomics data (*R*^2^*X*) or the MP grouping variable (*R*^2^*Y*) that can be explained by the model, and *Q*^2^ assesses each model’s predictive ability. Permutation tests and 5-fold cross-validation were used further to validate the models. Two-way mixed analysis of covariance was used to compare changes in metabolic health between MPs before and after the dietary intervention. *P* values of <0.05 were considered significant. Where there were significant differences between groups, Bonferroni post hoc tests were used to compare means between MPs and time interactions within MPs. Mean differences, 95% confidence intervals (CIs), and effect sizes (partial η^2^) are reported for post hoc tests. To account for false discovery rate, *P* values were adjusted using the Benjamini–Hochberg method when analyzing differences in nutrient intakes and metabolite concentrations between MPs (Tables 1 and 2**,** Supplemental Table 1). Differences in total-C outcomes were assessed between tertiles of food group change and MPs using boxplots and one-way repeated measure analysis of variance.

**TABLE 1:** Metabolites with top VIP Scores and baseline concentrations (μmol), split by metabolic phenotype

Variable	Model	VIP score	MP1 (*n* = 86)		MP2 (*n* = 110)		MP3 (*n* = 149)		*P* value
			Mean	SD	Mean	SD	Mean	SD	
Glutamate	MP1/MP2	2.27	64.29^1^	25.77	33.50^2^	16.73	44.12^3^	21.08	<0.001
PC aa C38:3	MP1/MP3, MP1/MP2	2.05	51.08^1^	12.02	42.44^2^	9.89	34.80^3^	7.61	<0.001
PC aa C40:5	MP1/MP3	1.91	10.03^1^	2.65	8.69^2^	2.12	6.89^3^	1.58	<0.001
PC ae C34:3	MP1/MP2	1.81	5.97^1^	1.53	8.27^2^	2.01	6.39^1^	1.46	<0.001
PC aa C34:1	MP1/MP3	1.79	218.61^1^	52.05	208.04^2^	45.99	160.41^3^	31.34	<0.001
PC aa C32:1	MP1/MP3	1.79	16.63^1^	7.30	12.91^2^	6.51	9.10^3^	3.64	<0.001
PC aa C36:3	MP1/MP3, MP1/MP2, MP2/MP3	1.77	130.74^1^	29.42	125.75^2^	22.22	99.34^3^	16.94	<0.001
C3	MP1/MP2	1.76	0.36^1^	0.12	0.27^2^	0.07	0.30^2^	0.09	<0.001
PC ae C34:2	MP1/MP2	1.73	9.15^1^	2.00	11.86^2^	2.43	9.27^1^	1.84	<0.001
PC aa C34:4	MP1/MP3	1.72	1.62^1^	0.49	1.43^2^	0.56	1.05^3^	0.36	<0.001
PC aa C38:5	MP1/MP3	1.66	51.95^1^	12.49	50.48^1^	10.72	38.76^2^	8.29	<0.001
PC aa C36:1	MP1/MP3, MP2/MP3	1.65	57.10^1^	16.51	55.57^1^	12.43	41.27^2^	9.04	<0.001
PC aa C36:2	MP2/MP3	1.61	229.59^1^	45.14	244.47^1^	36.93	190.85^2^	33.01	<0.001
Isoleucine	MP1/MP2	1.61	93.09^1^	21.99	74.65^2^	14.70	86.65^3^	20.13	<0.001
PC aa C40:6	MP1/MP3	1.60	24.83^1^	7.28	22.71^2^	6.66	17.27^3^	5.27	<0.001
SM C16:0	MP1/MP2, MP2/MP3	1.59	114.00^1^	18.80	132.94^2^	17.00	108.25^1^	15.29	<0.001
PC aa C36:5	MP1/MP3	1.58	28.52^1^	10.51	25.74^1^	11.78	17.53^2^	7.90	<0.001
SM (OH) C22:2	MP2/MP3	1.58	13.08^1^	3.06	15.76^2^	2.85	11.60^3^	2.37	<0.001
PC ae C36:0	MP2/MP3	1.57	0.71^1^	0.19	0.83^2^	0.16	0.62^3^	0.11	<0.001
PC ae C32:2	MP1/MP2	1.54	0.56^1^	0.11	0.69^2^	0.14	0.54^1^	0.10	<0.001
Leucine	MP1/MP2	1.53	191.35^1^	38.84	159.33^2^	30.19	178.78^2^	33.34	<0.001
PC aa C34:2	MP2/MP3	1.52	327.19^1^	60.04	337.25^1^	50.35	269.20^2^	43.68	<0.001
PC ae C36:1	MP2/MP3	1.49	6.33^1^	1.50	6.81^1^	1.30	5.18^2^	0.92	<0.001
SM (OH) C22:1	MP2/MP3	1.48	14.87^1^	3.17	15.72^1^	2.85	12.08^2^	2.35	<0.001
SM C16:1	MP2/MP3	1.47	17.24^1^	3.18	19.44^2^	3.15	15.30^3^	2.78	<0.001

Abbreviations: aa: diacyl; ae: acyl-alkyl; MP1, metabolic phenotype 1; MP2, metabolic phenotype 2; MP3, metabolic phenotype 3; PC, phosphatidylcholine; SM, sphingomyelin; VIP, variable importance in projection.

Superscript numbers (1 to 3) correspond to the statistical difference between phenotypes in post hoc pairwise comparison (Bonferroni).

Data presented in micromoles. The model corresponds to the PLS-DA model that included the VIP metabolite (MP1/MP2, MP1/MP3, or MP2/MP3). A univariate general linear model is used to measure significance. Pearson’s correlation is used to identify covariates. Covariates include sex and/or age (years) where a significant association with the dependent variable exists. A *P* value of <0.05 was considered significant. *P* values were adjusted for false discovery rate using Benjamini–Hochberg.

**TABLE 2:** Baseline mean daily food group intake and nutrient intake split by metabolic phenotype

	MP1 (*n* = 86)		MP2 (*n* = 110)		MP3 (*n* = 149)		*P* value
	Mean	SD	Mean	SD	Mean	SD	
Whole grains (g)	29.43	25.50	26.98	22.22	28.83	28.37	0.914
Fruit (g)	161.47	143.65	170.11	163.27	189.11	159.90	0.449
Vegetables (g)	151.39	73.95	158.84	71.66	152.23	80.30	0.661
Dark green vegetables (g)	27.56	25.52	39.71	35.92	33.97	34.09	0.109
Red/orange vegetables (g)	74.83	45.11	70.49	40.44	70.35	47.06	0.802
Legumes (g)	23.17	31.24	15.41	22.31	15.04	23.85	0.137
Nuts and seeds (g)	3.81	6.49	5.51	8.81	5.66	10.13	0.256
Total meat (g)	173.65	77.80	176.00	110.63	181.12	108.87	0.463
Fish (g)	21.60	29.08	23.02	27.79	16.87	25.56	0.202
Dairy (g)	212.71	168.58	211.85	137.45	237.12	166.82	0.234
Alcohol (g)	335.07^1^	485.38	221.13^2^	252.45	137.55^2^	255.95	0.005
Energy (kcal)	2293.93	651.87	2087.61	542.47	2122.43	615.74	0.091
Carbohydrate (g)	262.55^1^	82.13	222.11^2^	65.13	239.31^1^	81.66	0.017
Carbohydrate (%TE)	46.05^1^	7.62	42.64^2^	7.28	45.15^1^	7.37	0.019
Protein (g)	92.13	25.35	87.80	27.64	91.10	29.93	0.543
Protein (g/kg)	1.03^1^	0.33	1.20^2^	0.37	1.16^2^	0.42	<0.001
Protein (%TE)	16.34	3.36	17.05	3.64	17.47	4.62	0.295
Fat (g)	90.89^1^	34.05	88.00^2^	28.93	89.40^2^	33.06	0.011
Fat (%TE)	35.28^1^	6.20	37.58^2^	5.64	37.58^2^	6.21	0.033
Saturated fat (g)	34.93^1^	15.29	34.86^2^	13.37	34.83^2^	16.16	0.013
Saturated fat (% TE)	13.50	3.31	14.78	3.20	14.48	3.65	0.073
MUFA (g)	32.98	13.32	31.50	10.49	32.13	12.21	0.073
MUFA (%TE)	12.78	2.72	13.49	2.48	13.50	2.68	0.179
PUFA (g)	13.22	4.98	12.44	4.47	12.84	4.67	0.390
PUFA (% TE)	5.18	1.40	5.36	1.27	5.46	1.42	0.375
Fiber (g)	19.67	7.26	17.61	5.93	18.52	7.31	0.827
Sodium (mg)	2574.50	852.57	2277.03	903.01	2250.35	790.28	0.865
Dietary folate (μg)	238.69	104.06	226.91	73.34	239.33	99.94	0.396
Vitamin B12 (μg)	4.55	2.75	4.28	2.23	4.87	2.47	0.075
Vitamin C (mg)	71.13	61.45	90.95	45.84	80.66	60.89	0.245
Retinol (μg)	368.18^1^	238.32	399.12^2^	218.09	412.20^2^	463.24	0.008
Carotene (μg)	2518.04	2466.73	2772.30	2102.93	2545.20	2175.93	0.616
Vitamin D (μg)	3.13	2.68	3.18	2.19	2.91	2.10	0.237

Abbreviations: % TE, percent of total energy; MP1, metabolic phenotype 1; MP2, metabolic phenotype 2; MP3, metabolic phenotype 3.

Superscript numbers (1 and 2) correspond to the statistical difference between phenotypes in post hoc pairwise comparison (Bonferroni).

A univariate general linear model is used to measure significance. Pearson’s correlation is used to identify covariates. A *P* value of <0.05 is considered significant. Covariates include baseline energy (kcal/d), where a significant association with the dependent variable exists. *P* values were adjusted for false discovery rate using Benjamini–Hochberg.

## Results

### Participant characteristics

**FIGURE 1:**
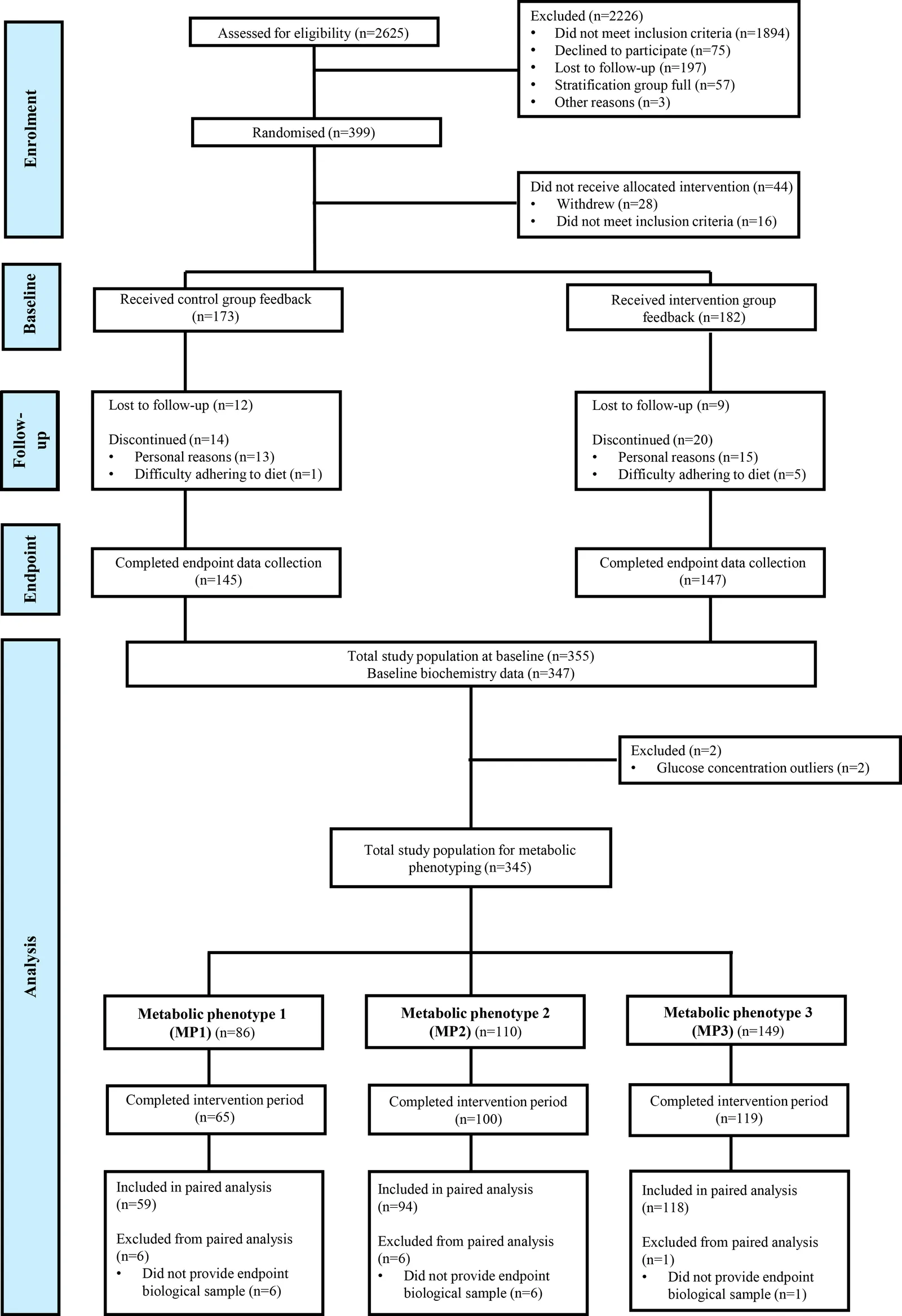
CONSORT flow diagram of participants included in phenotyping analysis. MP1, metabolic phenotype 1; MP2, metabolic phenotype 2; MP3, metabolic phenotype 3.

All participants with baseline metabolic health markers (*n* = 345) were included in the analysis (Figure 1). The sample population was 43% male and had a mean age of 41.82 ± 12.21 y. Metabolic phenotype 1 (MP1) (*n* = 86) was categorized as metabolically unhealthy with the highest concentrations of total-C (5.87 ± 0.90 mmol/L), TAGs (1.98 ± 0.54 mmol/L), LDL cholesterol (3.84 ± 0.85 mmol/L), glucose (5.47 ± 0.59 mmol/L), insulin (77.59 ± 47.41 pmol/L), and CRP (3.42 ± 3.73 mg/L). MP1 also had the highest anthropometric measurements, including BMI (31.05 ± 4.82 kg/m^2^), body fat percentage (36.05 ± 9.6%), waist circumference (101.33 ± 12.62 cm), and hip circumference (111.33 ± 9.03 cm). Metabolic phenotype 2 (MP2) (*n* = 110) was mostly female (75.5%) with the highest HDL cholesterol concentrations (1.91 ± 0.30 mmol/L) and lowest glucose (5.09 ± 0.41 mmol/L), insulin (38.80 ± 17.98 pmol/L), CRP (1.43 ± 2.12 mg/L), BMI (25.97 ± 4.01), waist circumference (86.08 ± 11.46 cm), and hip circumference (104.30 ± 8.42 cm). MP2 was characterized as the most anthropometrically healthy, although it had total-C concentrations (5.82 ± 0.85 mmol/L) and LDL cholesterol concentrations (3.53 ± 0.85 mmol/L) similar to the metabolically unhealthy MP1 (5.87 ± 0.90 mmol/L and 3.84 ± 0.85 mmol/L, respectively). Metabolic phenotype 3 (MP3) (*n* = 149) was the youngest cluster (38.23 ± 12.27 y) with the lowest total-C (4.43 ± 0.64 mmol/L), TAGs (0.81 ± 0.28 mmol/L), and LDL cholesterol (2.69 ± 0.62 mmol/L). Post hoc analysis revealed that MP1 had significantly (all *P <* 0.05) higher BMI, body fat percentage, TAGs, glucose, insulin, CRP, waist circumference, and hip circumference compared with other phenotypes (Table 3). MP1 and MP2 had significantly higher total-C and LDL cholesterol than MP3 (*P <* 0.05). MP2 had significantly higher HDL cholesterol than MP1 and MP3 (*P <* 0.05).

**TABLE 3:** Baseline demographics and metabolic health biomarkers split by metabolic phenotype

	MP1 (*n* = 86)		MP2 (*n* = 110)		MP3 (*n* = 149)		*P* value
	Count	%	Count	%	Count	%	
Intervention group							0.992
Intervention	44	51.2	56	49.1	77	51.7	
Control	42	48.8	54	48.3	72	48.3	

	Mean	SD	Mean	SD	Mean	SD	*P* value

Sex (% male)	53.5	—	24.5	—	51.0	—	<0.001
Age (y)	44.33^1^	11.15	44.72^1^	11.74	38.23^2^	12.27	<0.001
BMI (kg/m^2^)	31.05^1^	4.82	25.97^2^	4.01	27.57^3^	5.61	<0.001
Body fat (%)	36.05^1^	9.60	32.57^2^	8.36	30.68^3^	10.48	<0.001
Total-C (mmol/L)	5.87^1^	0.90	5.82^1^	0.85	4.43^2^	0.64	<0.001
TAGs (mmol/L)	1.98^1^	0.54	0.84^2^	0.29	0.81^2^	0.28	<0.001
HDL-C (mmol/L)	1.27^1^	0.24	1.91^2^	0.30	1.37^3^	0.21	<0.001
LDL-C (mmol/L)	3.84^1^	0.85	3.53^1^	0.85	2.69^2^	0.62	<0.001
Glucose (mmol/L)	5.47^1^	0.59	5.09^2^	0.41	5.16^2^	0.39	<0.001
Insulin (pmol/L)	77.59^1^	47.41	38.80^2^	17.98	51.91^3^	34.98	<0.001
CRP (mg/L)	3.42^1^	3.73	1.43^2^	2.12	1.71^2^	2.55	<0.001
Waist (cm)	101.33^1^	12.62	86.08^2^	11.46	90.26^3^	15.61	<0.001
Hip (cm)	111.33^1^	9.03	104.30^2^	8.42	106.98^2^	11.49	<0.001
SBP (mmHg)	125.26	11.67	117.13	11.86	117.75	14.29	0.183
DBP (mmHg)	83.34^1^	7.56	77.78^1,2^	7.53	77.54^2^	9.08	0.038
Physical activity (METS)	1875.23^1^	1905.87	2432.40^2^	2687.61	2165.8^1,2^	1800.18	0.030

Abbreviations: CRP, C-reactive protein; DBP, diastolic blood pressure; METS, Metabolic equivalent of task; MP1, metabolic phenotype 1; MP1, metabolic phenotype 1; MP2, metabolic phenotype 2; MP3, metabolic phenotype 3; SBP, systolic blood pressure; TAGs, triglycerides; Total-C, total cholesterol.

Superscript numbers (1–3) correspond to the statistical difference between phenotypes in post hoc pairwise comparison (Bonferroni).

Differences in the intervention group were compared using the chi-square test. Differences in continuous variables compared using univariate general linear model analysis of covariance. Pearson’s correlation is used to identify covariates. Covariates include sex, age (years), and/or BMI (kg/m^2^) where a significant association with the dependent variable exists. A *P* value of <0.05 is considered significant.

### Metabolomic profiles of the metabolic phenotypes

**FIGURE 2:**
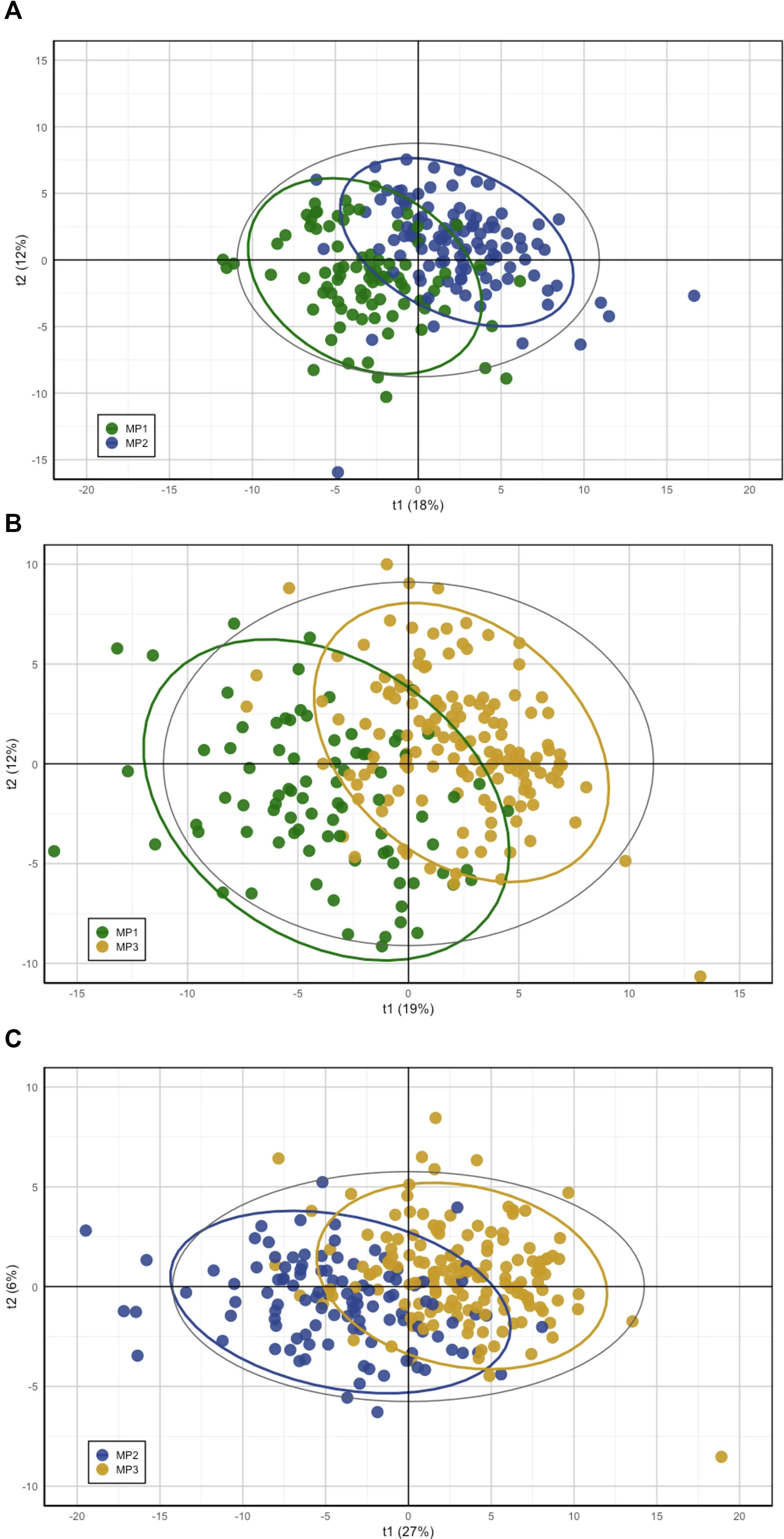
(A–C) PLS-DA scores plots, pairwise models of baseline metabolomic profiles of metabolic phenotypes. (A) Visit 1 MP1 and MP2 Scores (PLS-DA). (B) Visit 1 MP1 and MP3 Scores (PLS-DA). (C) Visit 1 MP2 and MP3 Scores (PLS-DA). Green represents MP1, blue represents MP2, and yellow represents MP3. (A) *R*^2^*X* = 0.30, *R*^2^*Y* = 0.49, *Q*^2^*Y* = 0.39. (B) *R*^2^*X* = 0.31, *R*^2^*Y* = 0.55, *Q*^2^*Y* = 0.49. (C) *R*^2^*X* = 0.33, *R*^2^*Y* = 0.49, *Q*^2^*Y* = 0.41. MP1, metabolic phenotype 1; MP2, metabolic phenotype 2; MP3, metabolic phenotype 3; PLS-DA, partial least squares discriminant analysis.

Pairwise comparisons of metabolomic profiles between MPs are presented in Figure 2. The differentiation in metabolomic profiles between MP1 and MP2 is presented in Figure 2A. The 2-component PLS-DA model (*R*^2^*X* = 0.30, *R*^2^*Y* = 0.49, *Q*^2^ = 0.39, *P =* 0.001) had 85.1% accuracy in cross-validation. The PLS-DA model for MP1 and MP3 was the most predictive (*R*^2^*X* = 0.31, *R*^2^*Y* = 0.55, *Q*^2^ = 0.49, *P =* 0.001) of the models (Figure 2B), showing 82.1% accuracy. PLS-DA of MP2 and MP3 resulted in a 2-component model (*R*^2^*X* = 0.33, *R*^2^*Y* = 0.49, *Q*^2^ = 0.41, *P =* 0.001) with 79.2% predictability accuracy in cross-validation (Figure 2C). There were 33 metabolites of interest (VIP > 1.2) differentiating MP1 and MP2, 32 differentiating MP1 and MP3, and 40 differentiating MP2 and MP3. All metabolites within the top 10 VIP scores for each model were significantly different across phenotypes at baseline (*P <* 0.001) (Table 1). Of metabolites with top VIP scores, there were 3 amino acids, 1 acylcarnitine, 4 SMs, and 17 PCs. Three metabolites were associated with 2 models (PC aa C38:3, PC aa C36:1, and SM C16:0), whereas PC aa C36:3 was associated with all 3 models. The full metabolomic profile for all MPs is presented in Supplemental Table 1.

### Mean daily food group and nutrient intakes

There were no differences in energy or food group intake between MPs at baseline (Table 2). Alcohol intake was significantly different between the phenotypes (*P =* 0.005), with MP1 having significantly higher intakes (335.07 ± 485.38 g) than MP2 (221.13 ± 252.45 g) and MP3 (137.55 ± 255.95 g). MP1 had the highest relative intake of carbohydrates (46.05 ± 7.62% total energy) and the lowest mean daily protein intake relative to body weight (1.03 ± 0.33 g/kg), percent of total energy from fat (35.28 ± 6.20%), and retinol intake (368.18 ± 238.32 μg) (all *P <* 0.05). There were no other significant differences in micronutrient intakes between the MPs.

### Changes in metabolic health biomarkers

There were 271 participants who provided blood samples at the end of the study period and were included in the repeated measures analysis (Table 4). There was a significant effect of time (*P =* 0.042) and time × group interaction (*P* = 0.001) for total-C during the study period. Post hoc analysis revealed significant decreases in total-C within MP1 (mean difference: −0.19 mmol/L; 95% CI: −0.33, −0.06, *P =* 0.013, partial *η*^2^ = 0.02) and MP2 (mean difference: −0.18 mmol/L; 95% CI: −0.29, −0.08, *P =* 0.001, partial *η*^2^ = 0.04). There was a significant time × group interaction (*P <* 0.001) for HDL cholesterol with significant decrease over time in MP2 (mean difference: −0.07 mmol/L; 95% CI: −0.11, −0.04, *P <* 0.001, partial *η*^2^ = 0.05) and increase over time in MP3 (mean difference: 0.03 mmol/L; 95% CI: 0.00, 0.06, *P =* 0.029, partial *η*^2^ = 0.02). The study resulted in a significant time × group interaction (*P =* 0.002) for TAG concentrations; post hoc analysis revealed a significant decrease within MP1 (mean difference: −0.19 mmol/L; 95% CI: −0.29, −0.08, *P =* 0.005, partial *η*^2^ = 0.03). There was a significant increase in MP3 with a mean difference of 0.08 mmol/L, 95% CI: 0.00, 0.15 (*P =* 0.046, partial *η*^2^ = 0.02). There was a significant time × group interaction for CRP (*P* = 0.043) and a significant decrease in MP1 (mean difference: −1.10 mg/L; 95% CI: −1.85, −0.35, *P =* 0.018, partial *η*^2^ = 0.02). After the study period, there was a significant decrease over time (*P =* 0.047) for waist circumference (mean difference: −0.71 cm, 95% CI: −1.24, −0.19, partial η^2^ = 0.03).

**TABLE 4:** Metabolic health biomarkers at baseline and endpoint split by the metabolic phenotype

	MP1 (*n* = 59)					MP2 (*n* = 94)					MP3 (*n* = 118)					2-way ANCOVA	
	Baseline		Endpoint		*P* value	Baseline		Endpoint		*P* value	Baseline		Endpoint		*P* value	*P* value	
	Mean	SD	Mean	SD		Mean	SD	Mean	SD		Mean	SD	Mean	SD		Time	Time × group
BMI (kg/m^2^)	30.51	4.76	30.18	4.72		25.78	4.01	25.71	4.18		27.04	5.59	26.84	5.6		0.572	0.216
Weight (kg)	90.80	16.47	89.77	15.98		74.08	12.27	73.88	12.73		81.05	19.98	80.45	19.76		0.164	0.482
Total-C (mmol/L)	5.92	0.88	5.74	0.83	0.013	5.81	0.86	5.64	0.94	0.001	4.42	0.68	4.49	0.80	0.146	0.042	0.001
LDL-C (mmol/L)	3.90	0.85	3.74	0.76		3.54	0.87	3.41	0.89		2.71	0.65	2.72	0.73		0.153	0.060
HDL-C (mmol/L)	1.27	0.25	1.28	0.27	0.684	1.90	0.30	1.83	0.32	<0.001	1.36	0.22	1.39	0.22	0.029	0.688	<0.001
TAGs (mmol/L)	1.90	0.52	1.72	0.60	0.005	0.82	0.25	0.88	0.33	0.121	0.78	0.27	0.86	0.36	0.046	0.864	0.002
Glucose (mmol/L)	5.49	0.53	5.42	0.69		5.06	0.39	4.99	0.42		5.19	0.37	5.12	0.40		0.447	0.941
Insulin (pmol/L)	71.61	46.18	72.12	48.7		37.67	18.54	38.15	19.59		49.33	30.2	49.57	29.85		0.522	0.843
CRP (mg/L)	3.40	4.05	2.35	2.33	0.018	1.24	1.86	1.24	1.50	0.743	1.50	2.24	1.99	3.49	0.340	0.905	0.043
Waist (cm)	99.93	12.05	98.96	12.30		85.53	11.66	84.88	11.85		89.81	16.02	89.25	15.93		0.047	0.731
Hip (cm)	109.55	8.97	109.13	9.08		103.77	8.53	103.78	8.80		106.31	11.64	103.37	11.64		0.430	0.109
SBP (mmHg)	126.97	11.10	124.4	10.50		117.18	11.67	116.65	13.20		118.32	14.78	117.93	15.44		0.468	0.188
DBP (mmHg)	83.38	7.95	83.16	7.83		77.48	7.56	77.04	7.69		77.96	9.32	78.66	9.38	-	0.618	0.374

Abbreviations: 2-way ANCOVA, 2-way mixed analysis of covariance; CRP, C-reactive protein; DBP, diastolic blood pressure; MP1, metabolic phenotype 1; MP2, metabolic phenotype 2; MP3, metabolic phenotype 3; RM ANCOVA, repeated measure analysis of covariance; SBP, systolic blood pressure; TAGs, triglycerides; Total-C, total cholesterol.

Pearson’s correlation is used to identify covariates. Covariates include sex, age (years), and/or BMI (kg/m^2^), where a significant association with the dependent variable exists. A *P* value of <0.05 is considered significant. Post hoc analysis within group when a significant time × group interaction.

### Changes in food group intakes relative to metabolic phenotype cholesterol response

**FIGURE 3:**
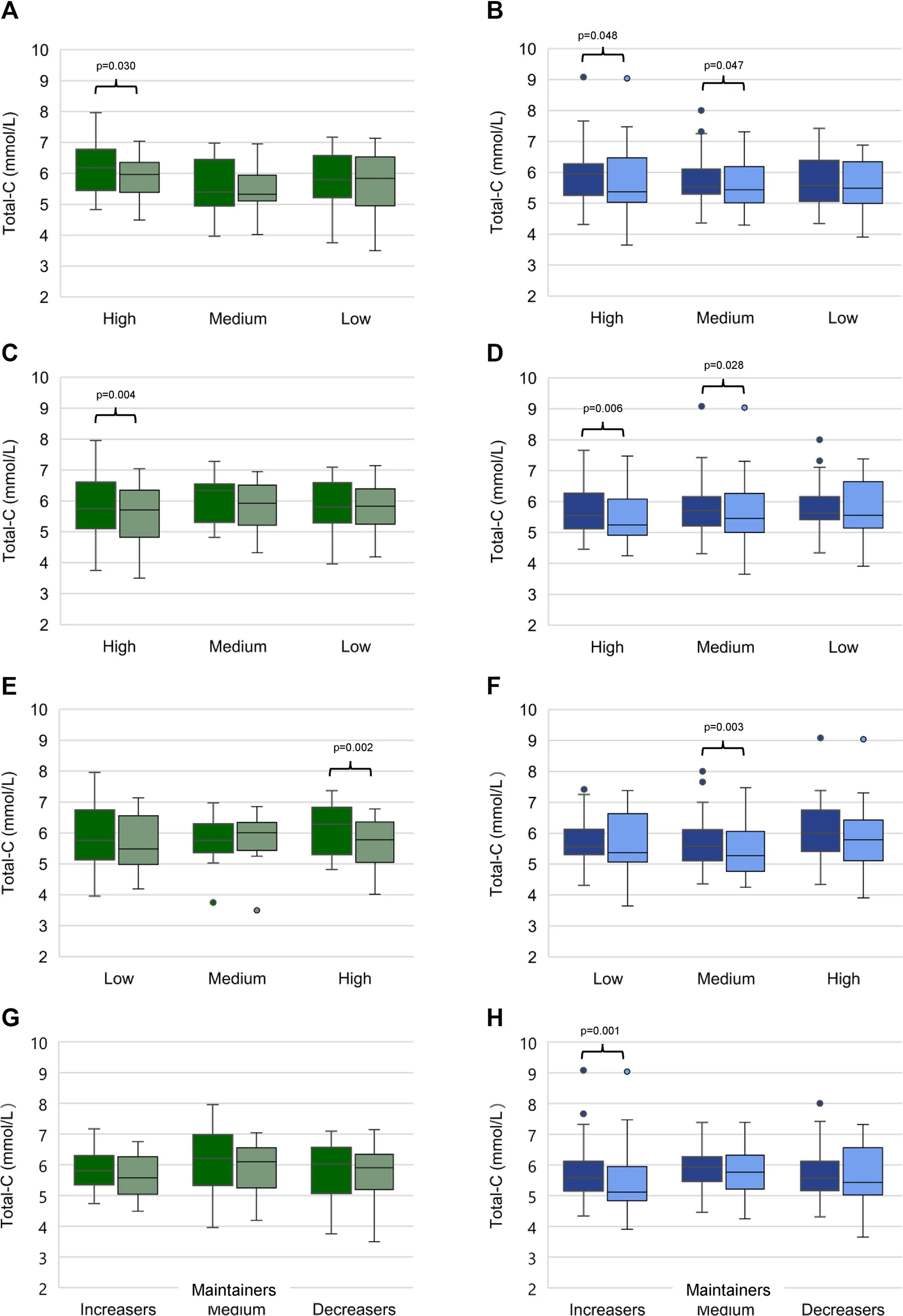
(A–H) Boxplots of total cholesterol at baseline and endpoint split by tertiles of change in food intake in MP1 and MP2. Boxplots on the left (green) represent MP1. Boxplots on the right (blue) represent MP2. Darker green boxes represent MP1 baseline. Lighter green boxes represent MP1 endpoint. Darker blue boxes represent MP2 baseline. Lighter blue boxes represent MP2 endpoint. (A, B) represent tertiles of change in fruit and vegetable intake. Low corresponds to the smallest increase, and high corresponds to the largest increase. (C, D) represent tertiles of change in whole grain intake. Low corresponds to the smallest increase, and high corresponds to the largest increase. (E, F) represent tertiles of change in meat intake. Low corresponds to the smallest decrease, and high corresponds to the largest decrease. Panels (G, H) represent tertiles of change in dairy intake. Significant (*P <* 0.05) time interactions were noted where applicable. Significance was measured with 1-way repeated measures analysis of variance. MP1, metabolic phenotype 1; MP2, metabolic phenotype 2; Total-C, total cholesterol.

To better show how MPs responded over time, further analysis was completed to assess if metabolic response was linked to food group intakes. Changes in food group intakes were calculated and split into tertiles. The relationship between the tertiles of food group change and total-C over time was examined within responsive MPs (MP1 and MP2). MP3 did not exhibit changes in total-C during the study and was not included in this analysis. Higher increases in intakes of whole grains and fruits and vegetables were associated with significant reductions in total-C in MP1 (*P* = 0.004) and MP2 (*P =* 0.006, *P =* 0.028) (Figure 3A–D). The highest change tertile of total meat intake (i.e., those in the tertile with the largest decrease) was associated with significant reductions in total-C within MP1 (*P* = 0.002) (Figure 3E). Moderate reduction in meat intake within MP2 was associated with a significant decrease in total-C (*P =* 0.003) (Figure 3F). Increasing dairy intake was associated with significant decreases in total-C in MP2 (*P* = 0.001) but not in MP1 (Figure 3G,H).

## Discussion

Exploratory analysis of the MyPlanetDiet sample population was able to identify three distinct MPs using four routinely measured metabolic biomarker concentrations. Four biomarkers (total-C, HDL cholesterol, TAGs, and glucose) were used for metabolic phenotyping based on their use and application in previous research [[8], [9], [10], [11],^19^]. The 3 MPs were significantly different in metabolic health markers such as anthropometry, inflammatory markers, blood pressure, and metabolomic profile at baseline. Two of the three MPs exhibited a positive response during the study, such as decreases in total-C. Further analysis revealed that high increases in fruit and vegetable and whole grain intakes during the study were associated with a decrease in total-C in the 2 responsive phenotypes. In one phenotype (MP1), those who decreased meat intake the most had lower total-C at the end of the study, whereas in the other responsive phenotype (MP2), those who increased dairy intake during the study, showed improvements in total-C. Our findings demonstrate the practicality of metabolic phenotyping for identifying distinct metabolic profiles and metabolic responses. To our knowledge, the present analysis is the first to examine metabolic phenotype–based response to a sustainable healthy diet and therefore offers new insights into creating a more effective personalization framework for sustainable healthy diets in the future.

Like previous publications, our analysis resulted in three MPs withone phenotype identified as the most “at-risk” with concentrations of total-C, TAGs, and glucose above healthy concentrations [9,11,19]. O’Donovan et al. [9], who first analyzed metabolic phenotypes using the criteria above, demonstrate that metabolic phenotyping can be effective at identifying “at-risk” groups who show other characteristics of disease risk, including high BMI, waist circumference, insulin concentrations, and markers of inflammation. The relationship between the metabolic phenotype and disease risk was further explored by Riedl et al. [19] using the German KORA F4 and FF4 cohorts. One MP, determined using the same criteria, had the highest concentrations of total-C, TAGs, and glucose, also the highest incidence of metabolic- and diet-related disease in a 7-y follow-up [19]. Although other research groups have used different phenotyping criteria (i.e., different cluster methodology, clustering based on more metabolites, or using different metabolites), the methodology used in the present analysis demonstrates how four routinely and easily measured biomarkers of metabolic health can be useful in identifying distinct phenotypes [[20], [21], [22]]. The use of metabolic phenotyping, both in terms of categorizing distinct phenotypes with different metabolic profiles and also in identifying disease risk, can be beneficial for future nutrition research. Identifying disease risk is an essential step in creating interventions and solutions to improve metabolic health.

Analysis of metabolomic profiles further differentiated the 3 MPs. It is well established that metabolomic profiles are strongly influenced by sex and age [23,24]. Interestingly, the strongest model in the present analysis was for MP1 and MP3, which both have ∼50:50 male:female ratios. The other models that included MP2 were not as predictive, despite MP2 being 75% female. Similarly, all baseline concentrations of top VIP metabolites for MP3 were significantly different from either MP1, MP2, or both, even when controlled for age. Our analysis therefore demonstrates that there is more to differentiating the phenotypes than just differences in age and sex distributions. When looking at specific metabolites, concentrations of amino acids glutamate, isoleucine, and leucine were highest among MP1 and lowest among MP2. Branched-chain amino acids, glutamate, and acylcarnitine (C3) are positively associated with increased BMI and prevalence of obesity [25]. Our findings support this association with MP1 having significantly higher BMI (31.05) compared with MP2 (25.97). Interestingly, when comparing VIP metabolites, MP1 and MP2 had the highest concentrations of PCs, although with clear differences emerging based on the chemical bonds between fatty acids and glycerol. In the five ae PCs differentiating the MPs in the PLS-DA analysis, the highest concentrations were consistently observed in MP2. Although MP1 had the highest concentrations in 10 of 12 aa PCs among VIP metabolites. Previously, differences in ester and ether bonds have been associated with differences in disease risk, even with the same fatty acid chain length and level of saturation [[26], [27], [28]]. Using metabolomic data from a large prospective cohort study, researchers reported a positive association between several diacyl PCs and risk of developing type 2 diabetes mellitus (T2DM), including several VIP metabolites from the present analysis (PC aa C32:1, C36:1, C38:3, and C40:5) [28]. In our analysis, MP1 had the highest concentrations of diacyl PCs and also the highest baseline concentrations of glucose and insulin. On the other hand, there was an inverse association between several ae PCs, including PC ae 34:3, as well as SM C16:1, and T2DM risk. PC ae 34:3 and SM C16:1 were both included in the list of VIP metabolites in the present analysis and were highest among MP2 [28]. Although genomic data were not available, genetic variation is likely influencing these differences [6]; for example, Gieger et al. [29] reported that PCs aa 34:2, aa 36:2, and ae 34:2 are positively associated with a specific genetic single-nucleotide polymorphism related to PUFA metabolism [29]. MP2 had the highest mean concentrations of these three metabolites, possibly indicating a genotype within MP2 that could influence both disease risk and also responsiveness to dietary interventions. Examining the difference in metabolomic profiles between the MPs demonstrates the complexity of factors that influence metabolic response.

After the identification and exploration of MPs, we sought to assess metabolic response to dietary change. Our previous analysis showed that there were no changes to metabolic biomarkers for the control or intervention groups, but previous literature has shown that MPs respond differently to dietary interventions [14,21,[30], [31], [32]]. This analysis revealed different responses between phenotypes for total-C, HDL cholesterol, TAGs, and CRP. For example, MP1 and MP2 decreased total-C, whereas no significant changes were seen in MP3. Post hoc analysis identified that MP1 and MP2 exhibited improvements in some metabolic health biomarkers, whereas MP3 was less responsive to dietary change. Therefore, applying a phenotyping approach identified significant improvements in some metabolic health markers for subgroups that were not seen in our previous analysis [14]. Identifying responders and nonresponders of dietary interventions is a stepping stone to understanding what factors predispose individuals to response or which external factors elicit response. As previously described, Kirwan et al. [7] analyzed criteria associated with positive cholesterol response to personalized nutrition feedback in the Food4Me RCT. In a model developed to classify responders and nonresponders, fatty acid concentrations, alcohol intake, and age were able to predict response in 90% of cases [7]. Similarly, O’Sullivan et al. [21] were able to identify a metabolic phenotype that demonstrated a positive metabolic response to vitamin D supplementation. Responders were vitamin D-deficient at baseline with several distinct metabolic profiles (e.g., higher resistin and adiponectin concentrations) that differentiated this cluster from other vitamin D-deficient nonresponders from other clusters [21]. In our analysis, MP1 and MP2 were significantly older with higher concentrations of total-C and LDL cholesterol compared with MP3, which showed no metabolic change in response to dietary change. Therefore, older participants with high baseline concentrations of total-C and LDL cholesterol were more likely to show a response to dietary change in the present study.

To better understand what drove metabolic differences over time, changes in mean daily food group intakes were examined between phenotypes. Those with the highest increases in daily fruit and vegetable and whole grain intakes had significant decreases in total-C in both MP1 and MP2. MP1 and MP2 responded differently to changes in intakes of total meat and dairy. Those who were in the tertile with the largest decrease in total meat intake decreased their total-C in MP1 but not in MP2. On the other hand, those with the largest increases in dairy intakes decreased total-C in MP2. Previous literature by O’Connor et al. [33] examining cholesterol response to dairy intake established that higher baseline total-C and HDL cholesterol concentrations were associated with greater decreases in total-C after a dairy-based intervention. In our analysis, MP2 had comparable baseline total-C with MP1, but significantly higher baseline concentrations of HDL cholesterol. It may be possible that the dairy matrix stimulates HDL-mediated cholesterol efflux in certain individuals, and those with higher HDL cholesterol concentrations have a greater capacity for total-C reduction [34]. However, discourse on the association between fat intake and metabolic health is ongoing as evidence suggests that dietary fat both increases and decreases metabolic disease risk in some individuals [35,36]. As previously stated, metabolic response to foods or food groups is mediated by a range of factors, including genetics, gut microbiome, metabolic health, and, importantly, other foods in the diet. For example, O’Sullivan et al. identified that habitual dark green vegetable intake was associated with metabolic response to omega-3 (n–3) supplementation in a 6-wk dietary intervention [37]. The authors suggest that the association could be attributed to reduced oxidative stress and increased lipid oxidation from high fruit and vegetable diets [37]. This work is one example of why it is important to consider how diets as a whole influence metabolic health. A combination of food groups makes up our habitual diets, and it is therefore important to establish how the body responds to multiple food groups and understand what synergistic mechanisms of foods may work together to support good health. Although such analysis is beyond the scope of the present analysis, to our knowledge, our findings are the first step in building future metabolic phenotype–based personalized nutrition strategies for sustainable healthy diets.

Our analysis uses metabolic health data, including anthropometry, clinical measurements, and serum biochemistry, and dietary assessments at multiple timepoints from the MyPlanetDiet study. A limitation of the present analysis is that data for other factors that influence interindividual variation and metabolic phenotype, such as genetics or gut microbiome composition, were not available. It is well established that multiple factors work together to regulate metabolic health, and therefore underpin the results of this analysis. At the same time, the findings presented here are derived from an exploratory secondary analysis and should therefore be considered hypothesis-generating. Accordingly, the statistical significance of these findings should be interpreted with caution. The dietary intake data used in the present analysis were generated from self-reported online 24-h recalls. Self-reported measures have inherent limitations that may impact the associations presented here in relation to change in food group intake and change in total-C. The clinical significance of these findings may be limited because of the short study duration (12 wk) and the study population. To better establish whether sustainable healthy diets could stimulate clinically meaningful change, future research should examine these relationships within a more targeted sample, such as individuals with obesity or those previously diagnosed with diet-related disease. MyPlanetDiet participants were randomly assigned into one of two groups of personalized dietary advice, resulting in dietary changes over time in both groups. To better understand metabolic response to a sustainable healthy diet, it may be important to use a traditional control group to differentiate metabolic changes related to sustainable healthy dietary intake. Furthermore, a traditional control group would allow for clearer differentiation as to whether the findings reported here are a result of phenotypic differences or a result of differences in behavior change within the sample population. However, MyPlanetDiet was the first study to test a whole-diet approach to a sustainable healthy diet. To our knowledge, this analysis is the first to examine metabolic phenotype–based response to a sustainable healthy diet.

In conclusion, *k*-means clustering using four commonly measured biomarkers resulted in 3 distinct MPs. The 3 MPs had significantly different clinical chemistry and anthropometry at baseline, but limited differences in mean daily intakes of food groups or nutrient intakes. MPs had significantly different metabolomic profiles at baseline, which offers further insight into what may be underpinning disease risk and metabolic response. Improving metabolic health is a key obstacle in the modern era, as is building more sustainable healthy diets. There is potential to establish novel dietary advice through metabolic phenotyping, which supports human and planetary health. The present analysis demonstrates that our personalized feedback for a more sustainable healthy diet elicited a positive metabolic response amongtwo of the three MPs. The analysis presented here was able to identify responses among subgroups that we had not been able to identify in our previous work [14]. Our personalized nutrition methodology, along with the findings from the present analysis, can be useful for future research to further explore metabolic phenotype–based personalized nutrition for sustainable healthy diets. To our knowledge, this analysis is ultimately the first step in embedding metabolic phenotyping into the existing MyPlanetDiet personalized framework. Future research should aim to support the individual and to utilize novel technologies to incorporate multiple layers of personalization into future dietary advice. The present study is one example of how interindividual variation will influence response to dietary advice and helps to demonstrate how proof-of-concept that personalized nutrition can improve health and sustainability through dietary change.

## Author contributions

The authors’ responsibilities were as follows – KPD, AMO’S: performed statistical analysis and wrote the paper; ERG, MEK, JVW, APN, SNM, AMO’S: designed the research; KPD, UML, LL, MCC: conducted the research; AMO’S: primarily responsible for final content; EA: contributed to revising the manuscript; and all authors: read, revised, and approved the manuscript.

## Data availability

Data described in the manuscript, code book, and analytic code will be made available on request pending corresponding author approval, ethical approval, and data management agreements.

## Funding

The SuHe Guide project is funded through the Department of Agriculture, Food and the Marine (DAFM) (info@agriculture.gov.ie)/Food Institutional Research Measure (FIRM) (grant number: 2019R546), and the Department of Agriculture, Environment and Rural Affairs (DAERA) (daera.helpline@daera-ni.gov.uk) (grant number 19/R/546). DAFM and DAERA had no role in the design, analysis, or writing of this article.

## Declaration of Generative AI and AI-assisted technologies in the writing process

The author(s) declare that no generative AI or AI-assisted technologies were used in the writing of this manuscript.

## Conflict of interest

The authors report no conflicts of interest.
