# Supplementary material for: Metabolic Phenotyping Identifies Responders to Sustainable Healthy Diets: Analysis from the MyPlanetDiet Randomized Controlled Trial
Source: Curr Dev Nutr. 2026 Aug 10;10(9):109510. doi: 10.1016/j.cdnut.2026.109510 (PMC13571452; doi:10.1016/j.cdnut.2026.109510)
Supplement: Supplementary file 1 [file mmc1.pdf]

Davies *et al.* Metabolic phenotyping identifies responders to sustainable healthy diets: analysis from the MyPlanetDiet randomised controlled trial

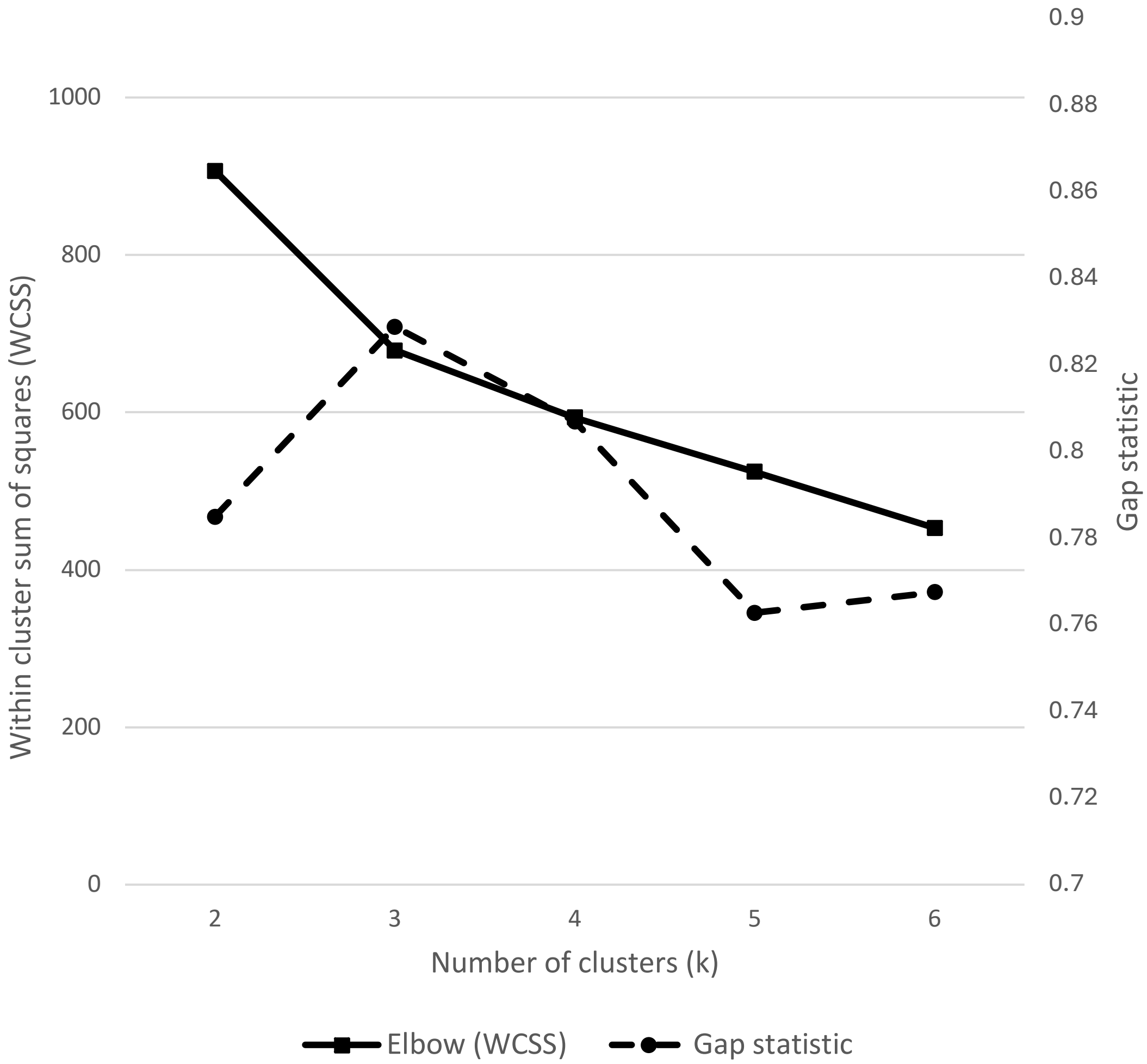

Supplementary Figure 1: Cluster validation metrics

k-means cluster validation using elbow method (right axis) and gap statistic (left axis). Solid line represents within cluster sum of squares (WCSS) (elbow method). Dotted line represents gap statistic.

Supplementary Table 1: Baseline concentrations of metabolites, split by metabolic phenotype

	MP1 (n=86)		MP2 (n=110)		MP3 (n=149)		
	Mean	SD	Mean	SD	Mean	SD	p-value
Serum amino acid concentrations							
Alanine	424.67	84.47	366.01	76.37	372.09	81.95	<0.001
Arginine	114.29	19.84	114.06	19.53	112.67	18.55	0.785
Asparagine	44.95	7.50	48.31	8.20	47.76	7.87	0.001
Aspartate	9.70	4.05	7.92	3.56	8.58	3.92	0.008
Citrulline	31.74	6.50	35.22	7.49	33.26	7.71	0.005
Glutamine	703.63	111.97	726.00	94.18	719.39	89.61	0.027
Glutamate	64.29	25.77	33.50	16.73	44.12	21.08	<0.001
Glycine	256.03	88.56	283.03	76.49	270.50	62.36	0.286
Histidine	98.24	12.38	98.44	11.45	99.86	12.09	0.579
Isoleucine	93.09	21.99	74.65	14.70	86.65	20.13	<0.001
Leucine	191.35	38.84	159.33	30.19	178.78	33.34	<0.001
Lysine	298.64	33.79	285.63	32.86	292.09	32.68	0.161
Methionine	28.32	5.94	26.70	5.23	28.65	5.88	0.627
Ornithine	91.78	19.36	89.36	16.62	90.36	17.30	0.803
Phenylalanine	96.47	13.04	92.00	12.13	97.66	15.83	0.138
Proline	196.37	51.06	168.10	40.62	195.56	53.03	0.005
Serine	117.28	19.99	128.98	22.80	130.64	22.36	<0.001
Threonine	127.62	26.93	128.91	34.78	132.01	27.49	0.527
Tryptophan	75.21	13.75	69.95	11.74	73.33	13.60	0.422
Tyrosine	127.62	26.93	128.91	34.78	132.01	27.49	0.005
Valine	317.20	64.27	282.16	53.17	299.71	57.21	0.023
Acylcarnitines							
Free carnitine (C0)	34.40	6.66	31.16	6.07	32.62	6.77	0.093
Acylcarnitine (C2)	5.90	1.67	6.17	1.97	6.38	1.99	0.192
Propionyl-carnitine (C3)	0.36	0.12	0.27	0.07	0.30	0.09	<0.001
Butyrylcarnitine (C4)	0.20	0.10	0.16	0.07	0.19	0.11	0.045
Decanoylcarnitine (C10)	0.24	0.11	0.31	0.27	0.27	0.14	0.040
Palmitoylcarnitine (C16)	0.11	0.03	0.11	0.03	0.10	0.02	0.012
Oleoylcarnitine (C18:1)							0.137
Biogenic amines							
ADMA (asymmetric dimethylarginine)	0.46	0.06	0.46	0.06	0.46	0.08	0.876
alpha-AAA (alpha-aminoadipic acid)	0.92	0.37	0.69	0.23	0.78	0.29	<0.001
Creatinine	74.87	15.98	72.64	13.50	75.92	16.18	0.178
Kynurenine	2.64	0.61	2.35	0.52	2.45	0.60	0.026
Putrescine	0.20	0.08	0.21	0.08	0.21	0.10	0.509
Sarcosine	1.22	0.42	1.26	0.47	1.32	0.45	0.040

SDMA (symmetric dimethylarginine)	0.41	0.08	0.43	0.09	0.41	0.08	0.026
Serotonin	0.64	0.42	0.73	0.42	0.72	0.40	0.271
t4-OH-Pro	9.49	4.89	9.36	4.85	10.57	6.17	0.292
Taurine	76.45	22.82	76.68	25.41	81.20	27.81	0.411
Lysophosphatidylcholines							
LYSO PC a C14:0	4.85	1.39	4.46	1.55	3.81	1.12	<0.001
LYSO PC a C16:0	64.47	12.95	69.34	12.95	61.30	11.48	<0.001
LYSO PC a C16:1	2.17	0.57	2.10	0.55	1.84	0.49	<0.001
LYSO PC a C17:0	1.23	0.41	1.51	0.44	1.27	0.39	<0.001
LYSO PC a C18:0	19.80	5.17	22.96	5.45	19.04	4.24	<0.001
LYSO PC a C18:1	14.06	3.97	18.60	5.63	15.92	4.65	<0.001
LYSO PC a C18:2	20.26	6.49	28.50	10.68	26.14	9.78	<0.001
LYSO PC a C20:3	1.81	0.54	1.82	0.59	1.71	0.61	0.009
LYSO PC a C20:4	4.39	1.36	4.97	1.73	4.85	1.71	<0.001
LYSO PC a C28:0	0.14	0.03	0.15	0.30	0.13	0.03	<0.001
Phosphatidylcholines							
PC aa C28:1	2.96	0.69	3.23	0.65	2.42	0.55	<0.001
PC aa C30:0	4.27	1.54	4.38	1.80	2.97	0.88	<0.001
PC aa C32:0	11.41	2.05	11.93	2.13	9.51	1.50	<0.001
PC aa C32:1	16.63	7.30	12.91	6.51	9.10	3.64	<0.001
PC aa C32:2	4.41	1.59	3.98	1.62	2.83	1.12	<0.001
PC aa C32:3	0.40	0.10	0.42	0.10	0.32	0.80	<0.001
PC aa C34:1	218.61	52.05	208.04	45.99	160.41	31.34	<0.001
PC aa C34:2	327.19	60.04	337.25	50.35	269.20	43.68	<0.001
PC aa C34:3	15.99	4.82	15.62	4.39	11.43	3.14	<0.001
PC aa C34:4	1.62	0.49	1.43	0.56	1.05	0.36	<0.001
PC aa C36:0	1.89	0.80	2.48	1.01	1.82	0.66	<0.001
PC aa C36:1	57.10	16.51	55.57	12.43	41.27	9.04	<0.001
PC aa C36:2	229.59	45.14	244.47	36.93	190.85	33.01	<0.001
PC aa C36:3	130.74	29.42	125.75	22.22	99.34	16.94	<0.001
PC aa C36:4	167.28	36.67	160.30	38.51	130.38	26.92	<0.001
PC aa C36:5	28.52	10.51	25.74	11.78	17.53	7.90	<0.001
PC aa C36:6	1.09	0.42	1.01	0.36	0.69	0.27	<0.001
PC aa C38:0	2.25	0.64	2.84	0.78	2.17	0.59	<0.001
PC aa C38:1	0.62	0.40	1.03	0.56	0.80	0.38	<0.001
PC aa C38:3	51.08	12.02	42.44	9.89	34.80	7.61	<0.001
PC aa C38:4	109.25	23.53	93.74	22.40	78.66	17.38	<0.001
PC aa C38:5	51.95	12.49	50.48	10.72	38.76	8.29	<0.001
PC aa C38:6	65.05	20.15	66.02	18.21	49.17	14.58	<0.001
PC aa C40:2	0.20	0.06	0.23	0.07	0.20	0.06	<0.001
PC aa C40:3	0.42	0.10	0.46	0.10	0.38	0.07	<0.001
PC aa C40:4	2.75	0.63	2.61	0.64	2.16	0.44	<0.001
PC aa C40:5	10.03	2.65	8.69	2.12	6.89	1.58	<0.001
PC aa C40:6	24.83	7.28	22.71	6.66	17.27	5.27	<0.001
PC aa C42:0	0.44	0.10	0.52	0.12	0.42	0.11	<0.001

PC aa C42:1	0.15	0.05	0.18	0.04	0.15	0.04	<0.001
PC aa C42:2	0.18	0.06	0.19	0.05	0.16	0.05	<0.001
PC aa C42:4	0.12	0.04	0.13	0.03	0.11	0.03	<0.001
PC aa C42:5	0.27	0.07	0.27	0.07	0.21	0.06	<0.001
PC ae C30:0	0.31	0.09	0.36	0.09	0.28	0.07	<0.001
PC ae C30:2	0.09	0.02	0.10	0.02	0.08	0.02	<0.001
PC ae C32:1	2.17	0.38	2.58	0.47	2.11	0.37	<0.001
PC ae C32:2	0.56	0.11	0.69	0.14	0.54	0.10	<0.001
PC ae C34:0	1.32	0.41	1.40	0.37	1.05	0.25	<0.001
PC ae C34:1	8.43	1.85	9.36	1.74	7.38	1.21	<0.001
PC ae C34:2	9.15	2.00	11.86	2.43	9.27	1.84	<0.001
PC ae C34:3	5.97	1.53	8.27	2.01	6.39	1.46	<0.001
PC ae C36:0	0.71	0.19	0.83	0.16	0.62	0.11	<0.001
PC ae C36:1	6.33	1.50	6.81	1.30	5.18	0.92	<0.001
PC ae C36:2	12.28	2.97	14.57	3.18	11.25	2.36	<0.001
PC ae C36:3	6.58	1.27	8.10	1.59	6.30	1.15	<0.001
PC ae C36:4	14.90	3.13	16.73	3.69	14.48	3.11	<0.001
PC ae C36:5	9.60	2.26	10.98	2.79	9.05	2.29	<0.001
PC ae C38:0	2.50	0.69	2.68	0.69	1.88	0.54	<0.001
PC ae C38:1	0.26	0.19	0.35	0.17	0.26	0.15	<0.001
PC ae C38:2	1.28	0.30	1.49	0.30	1.15	0.24	<0.001
PC ae C38:3	3.08	0.68	3.17	0.59	2.53	0.50	<0.001
PC ae C38:4	11.21	2.20	11.37	2.35	10.52	1.85	<0.001
PC ae C38:5	13.31	2.30	15.49	2.86	13.18	2.36	<0.001
PC ae C38:6	5.96	1.41	7.06	1.73	5.42	1.25	<0.001
PC ae C40:1	1.08	0.25	1.26	0.26	0.93	0.22	<0.001
PC ae C40:2	1.39	0.32	1.52	0.29	1.20	0.23	<0.001
PC ae C40:3	0.63	0.11	0.72	0.11	0.59	0.08	<0.001
PC ae C40:4	1.40	0.24	1.62	0.26	1.41	0.25	<0.001
PC ae C40:5	2.21	0.39	2.58	0.43	2.17	0.37	<0.001
PC ae C40:6	3.65	0.90	4.35	1.09	339.00	0.79	<0.001
PC ae C42:2	0.46	0.11	0.53	0.10	0.41	0.08	<0.001
PC ae C42:3	0.61	0.14	0.73	0.15	0.56	0.13	<0.001
PC ae C42:4	0.55	0.14	0.68	0.15	0.60	0.13	<0.001
PC ae C42:5	1.33	0.23	1.60	0.30	1.45	0.27	<0.001
PC ae C44:3	0.07	0.02	0.08	0.03	0.06	0.02	<0.001
PC ae C44:4	0.31	0.12	0.38	0.09	0.34	0.12	<0.001
PC ae C44:5	1.36	0.38	1.67	0.45	1.49	0.39	<0.001
PC ae C44:6	0.91	0.21	1.11	0.26	0.96	0.21	<0.001
Sphingolipids							
SM (OH) C14:1	6.84	1.63	8.09	1.60	6.26	1.30	<0.001
SM (OH) C16:1	3.76	0.90	4.33	0.86	3.45	0.71	<0.001
SM (OH) C22:1	14.87	3.17	15.72	2.85	12.08	2.35	<0.001
SM (OH) C22:2	13.08	3.06	15.76	2.85	11.60	2.37	<0.001
SM (OH) C24:1	1.48	0.42	1.61	0.43	1.26	0.37	<0.001

SM C16:0	114.00	18.80	132.94	17.00	108.25	15.29	<0.001
SM C16:1	17.24	3.18	19.44	3.15	15.30	2.78	<0.001
SM C18:0	27.39	5.17	28.75	4.93	23.38	4.85	<0.001
SM C18:1	12.88	3.20	14.04	2.72	11.26	2.54	<0.001
SM C20:2	0.38	0.09	0.44	0.10	0.34	0.08	<0.001
SM C24:0	24.18	4.39	24.58	4.39	19.68	3.65	<0.001
SM C24:1	58.61	12.02	66.91	10.79	53.81	9.28	<0.001
SM C26:0	0.13	0.07	0.14	0.06	0.11	0.06	0.020
SM C26:1	0.26	0.09	0.30	0.09	0.24	0.07	<0.001
Sugars							
Hexose	5062.72	730.35	4740.63	572.22	4854.22	520.03	0.008

MP1, Metabolic phenotype 1. MP2, metabolic phenotype 2. MP3, metabolic phenotype 3. SD, standard deviation. PC, phosphatidylcholine. aa: diacyl. ae: acyl-alkyl. SM, sphingomyelin. Data presented in micromoles. Univariate general linear model used to measure significance. Pearson's correlation used to identify covariates. P-value <0.05 considered significant. P-values were adjusted for false discovery rate using Benjamini–Hochberg.
